# Detection and quantification of *Anaplasma phagocytophilum* and *Babesia* spp. in *Ixodes ricinus* ticks from urban and rural environment, northern Poland, by real-time polymerase chain reaction

**DOI:** 10.1007/s10493-015-9887-2

**Published:** 2015-02-27

**Authors:** Joanna Stańczak, Stella Cieniuch, Anna Lass, Beata Biernat, Maria Racewicz

**Affiliations:** Department of Tropical Parasitology, Institute of Maritime and Tropical Medicine, Medical University of Gdańsk, 9B Powstania Styczniowego Street, 81-519 Gdynia, Poland

**Keywords:** Qualitative real-time PCR, qPCR, *Anaplasma phagocytophilum*, *Babesia* spp., *Ixodes ricinus*, Urban and rural environment, Poland

## Abstract

*Anaplasma phagocytophilum* and *Babesia* spp. are emerging tick-borne pathogens which can threaten human health. A duplex real-time PCR and qPCRs with primers and probes targeting 97 and 116 bp fragments of 16S rRNA and 18S rRNA genes, respectively, were used for qualitative and quantitative detection of both pathogens in *Ixodes ricinus* ticks. Altogether 1875 ticks (1084 adults and 791 nymphs) were collected from rural and urban habitats in northern Poland. Of them, at least 0.9 % were found to be infected with *A. phagocytophilum* while 2.5 % with *Babesia* spp. A comparison of the infection rates by the tick stage, the type of area, the collection site, habitats of different tick density and by the month of collection was done. The prevalence of pathogens was significantly lower in nymphs than in adult ticks (*p* = 0.02) and in rural areas than in urban areas (*p* = 0.007). Four different 16S rRNA gene variants of *A. phagocytophilum* were determine, however none of them showed 100 % identity with compared sequences isolated from human patients. The dominant *Babesia* species was *B. venatorum.* Results of qPCRs with circular and linearized forms of plasmids used as the standards showed significant difference in the pathogen loads (*p* = 0.001). The copy numbers of *A. phagocytophilum* and *Babesia* spp. estimated from the linear plasmids were 28.7 and 5.1 times lower, respectively, when compared with their circular forms, and were accepted as more reliable. The average number of copies of 16S rRNA gene of *A. phagocytophilum* in the positive *I. ricinus* samples were 3.39 × 10^5^ ± 6.09 × 10^5^. The mean copy number of 18S rRNA gene of *Babesia* spp. was ~2.55 × 10^5^ ± 1.04 × 10^6^. We confirmed the presence of *A. phagocytophilum* and *Babesia* spp. in *I. ricinus* in both rural and urban environments. The determined low infection rates suggests, however, that the risk for local population and tourists to acquire infection is also low. Moreover, we confirmed recent findings that serious overestimation by circular plasmid DNA makes it less suitable as a standard and that the linear standards should be recommended for qPCR.

## Introduction

*Ixodes ricinus* (Acari: Ixodidae), being the most common tick species in Europe, readily attack people and as a vector of different pathogens can threaten their health and even life. In Poland, it is the primary vector of *Borrelia burgdorferi* sensu lato, with infection rate even up to 58.8 % (Siński et al. 1994). Lyme borreliosis, caused by this spirochete, is the most commonly diagnosed tick-borne disease, with over 12,000 human cases reported in Poland in 2013 (33.12/100,000) (www.pzh.gov.pl). *I. ricinus* also acts as reservoir hosts for *Rickettsia* spp. of the spotted fever group rickettsiae, of which *R. helvetica* is the dominant species within its populations with prevalence ranging from 1.3 to 11.4 % (Chmielewski et al. 2009; Stańczak et al. 2008). Although *R. helvetica* has been isolated from patients with acute perimyocarditis, unexplained febrile illness or nonspecific fevers (Fournier et al. 2000) its pathogenic potential still remains unclear. In Poland, no clinical cases due to *R. helvetica* have been diagnosed so far. Less frequently *I. ricinus* was found to be infected with the other rickettsial pathogen, *Anaplasma phagocytophilum,* an obligate intracellular pathogen that parasitises the granulocytes of wild and domestic animals and man, as well as with protozoans of the genus *Babesia,* which infect and destroy red blood cells of infected vertebrate hosts. In Poland, the infection levels with these two pathogens in ticks varied between 0.6 % (Welc-Falęciak et al. 2012) and 38.5 % (Stańczak et al. 2002), and from 0.6 % (Siński et al. 2006; Stańczak et al. 2004) to 13.3 % (Skotarczak et al. 2003), respectively. Although the incidence of human granulocytic anaplasmosis (HGA) in the United States appears to increase annually (Dumler et al. 2007), symptomatic infections in Europe seem to be rare (Blanko and Oteo 2002). Also human babesiosis is more frequently diagnosed in the United States than in Europe, where only about 40 cases of *B. divergens* infection have been documented so far, mostly in France, Ireland, and Great Britain (Vannier and Krause 2009). However, in the last decade, three cases of infection with a new *Babesia* species, named *B. venatorum* (previously referred to as EU1) were reported from Austria, Italy and Germany (Herwaldt et al. 2003; Haselbarth et al. 2007). Although only about a dozen of confirmed clinical cases of HGA (Grzeszczuk et al. 2004, 2006; Tylewska-Wierzbanowska et al. 2001; Hermanowska-Szpakowicz et al. 2004) and only one indigenous case of human babesiosis (Przybylińska et al. 2004) have been diagnosed in Poland so far, both diseases are treated as emerging in our country.

Taking into consideration that climate change and the human pressure on the environment may result in increased prevalence of ticks and tick-borne infections (Gray et al. 2009; Ruiz-Fonz et al. 2012), and that the risk of acquiring such infections by humans is closely related to the prevalence of pathogens in active ticks, the purpose of our study was to investigate the prevalence of *A. phagocytophilum* and *Babesia* spp. potentially pathogenic for humans (*B. divergens, B. venatorum*) in *I. ricinus* ticks collected in urban and rural areas in northern Poland. For simultaneous qualitative detection and identification of target pathogens we evaluated a TaqMan-based duplex real-time PCR. Moreover, we made attempts to estimate the load of pathogens in ticks. Quantification of *A. phagocytophilum* and *Babesia* spp. in tick samples was performed in single reactions as absolute qPCR by comparison of the fluorescence signals of the sample with those of a standard curve by using circular and linearized standard plasmid DNAs.

To assess the risk of infection in the human population and for a better understanding of the circulation of these pathogens in nature, qualitative and quantitative analyses were done in relation to the tick stage, habitat, tick densities and collection months.

## Materials and methods

### Study area

The cities of Gdańsk, Sopot and Gdynia, Pomeranian voivodeship, northern Poland, forms a conurbation called the Tri-City. Within their borders is a large part of the Tri-City Landscape Park (TCLP), which covers the area of about 20 0000 ha and consists of two forest complexes, divided by urbanized city districts. Its location in the close vicinity of a large agglomeration makes the Park an extremely valuable destination for tourism and leisure among Tri-City residents. The TCLP is situated on the north-eastern edge of the Kashubian Lake District (KLD), one of the largest forest areas in Poland, a region visited by many tourists and popular choice among city dwellers to locate their summer houses. The most common tree species in both deciduous and mixed forests is the European beech (*Fagus sylvatica*).

**Fig. 1:**
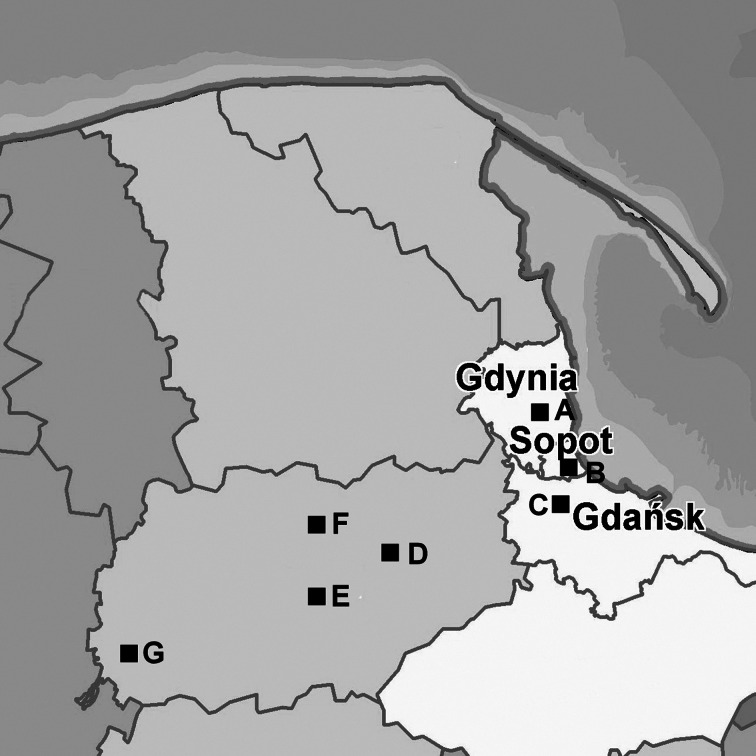
Locations of sampling sites. *A*, *B*, *C*—the Tri-City agglomeration; *D*, *E*, *F*, *G*—the Kashubian Landscape Park; Pomeranian voivodeship, northern Poland

Questing nymphs and adult *I. ricinus* ticks were collected from lower vegetation and a litter in spring-summer (April to August) seasons during 2009–2010 in seven variable localities (Fig. 1).

Three heavily frequented sites were located within the administrative borders of the Tri-City agglomeration. Two of them, site A in Gdynia-Kolibki (54°28′5.92″N, 18°27′18.98″E) and site C in Gdańsk–Jaśkowa Dolina (54°21′57.32″N, 18°35′11.24″E), were situated along walking paths on the edge of the municipal forests of the TCLP, a popular area for hiking, biking, horse riding and dog walking. The third site, B was located in intra-city Sopot-Karlikowo (54°25.57′55″N, 18°34′40.98″E), in a wasteland behind the sport hall with a small grove of deciduous trees and clusters of grey willow (osier). The place was used by the locals as a walking area for dogs.

Four other sites were situated in the Kashubian Landscape Park (KLP), being a part of the KLD. Site D—Kartuzy (54°20′34.13″N, 18°10′28.54″E), in a forested area outside the city borders, on the edge of mixed coppice forest and wet meadows. Site E—Borzestowska Huta (54°18′24.11″N, 18°02′48.48″E), on the edge of a mixed forest dominated by pine trees, in a rural area in the vicinity of the Lake Raduńskie. Site F—Mirachowo (54°24′11.16″N, 18°01′31.0″E), along the side of a road passing through a mixed forest of with a predominance of beech trees. Site G—Sulęczyno (54°15′08.01″N, 17°47′31.67″E), on the edge of a young forest of mixed stand in a recreational area by the Lake Węgorzyno; affected by high anthropopression during the spring—autumn season.

### Tick collection

To collect active ticks a standard flagging method was used. In the laboratory, ticks were killed by rapid immersion in a hot water, identified to species level by using the appropriate taxonomic key (Siuda 1993), separated by developmental stage and then preserved in 70 % ethanol until analysed.

### DNA extraction

All ticks were analysed individually. Extraction of nucleic acids from the ticks was done by lysis in ammonium hydroxide (NH_4_OH) (Rijpkema et al. 1996). The obtained lysates were stored at −20 °C until use as templates for the real time PCR.

### *Anaplasma phagocytophilum*-specific and *Babesia*-specific primers and probes

The pairs of forward and reverse primers and probes sequences, designed to be specific for *A. phagocytophilum* 16S rRNA and for *Babesia* spp. 18S rRNA genes by using Beacon Designer^™^ software, are shown in Table 1.

**Table 1:** Names and sequences of the duplex real-time PCR primers and probes

Pathogen	Target	Name	Sequence (5′–3′)	Amplicon size (bp)
*Anaplasma phagocytophilum*	16S rRNA	Aph16S-f	CGGGAGAGGATAGCGGAATTC	97
		Aph16S-r	CGTCAGTACCGGACCAGATAG	
		Aph16S-p	CY5-CGCCTTCGCCACTGGTGTTCCTCC-BHQ3	
*Babesia* spp.	18S rRNA	Bab18S-f	CATGAACGAGGAATGCCTAGT ATG	116
		Bab18S-r	CCGAATAAT TCA CCG GAT CAC TC	
		Bab18S-p	FAM-AAGTCATCAGCTTGTGCAGATTAC GTCCCT-BHQ1	

Primers specific for *A. phagocytophilum*, Aph16S-f and Aph16S-r, produce a 97 bp amplicon with a TaqMan probe Aph16Sp labelled at the 5′ and 3′ ends with the cyanine dye CY5^®^ and “Black Hole Quencher” BHQ3, respectively. Primers for the *Babesia* spp., Bab18S-f and Bab18S-r amplify a 116-bp fragment of the gene. A TaqMan probe identified as Bab18S-p, was labelled at the 5′ and 3′ ends with FAM (5-carboxyfluorescein) and BHQ1, respectively. This primer/probe combination was predicted to amplify the DNA of “large” *Babesia* species (*B. divergens*, *B. venatorum*, *B. capreoli*, *B. canis* etc.), but not *B. microti.* Both probes were HPLC purified.

### Optimized conditions for duplex real-time PCR

PCR was performed in a duplex format with a total reaction volume of 20 µL, by using the Real-Time 2× PCR Mastermix Probe (A&A Biotechnology, Gdynia, Poland) in a Mx3005P Real-Time QPCR System (Stratagene, Cal, USA). Optimal reaction conditions used 10 µL of the Mastermix Probe, 0.4 µL of each primer (10 µM), 0.2 µL of each probe (10 µM), 6 µL of water and 2 µL of template DNA extracted from the tick.

Negative and positive controls were included in all runs. *A. phagocytophilum*-positive and *Babesia*-positive controls were constructed by cloning the 97 and 116-bp PCR amplicons, respectively, into a circular pJet1.1 plasmids (Fermentas, USA) which were transformed into competent TOP10F’ *Escherichia coli* (Invitrogen). Then plasmids were extracted by using Plasmid Mini commercial kit (A&A Biotechnology, Gdynia, Poland). Concentrations of plasmids were measured with spectrophotometric method (NanoDrop 1000 spectrophotometer, Thermo Scientific, USA). A 1 µL of each control template was added to the reaction mixture. For qualitative assay, the circular plasmid DNA was used due to its high stability and reproducibility.

Cycling conditions included an initial activation of the *Taq* DNA polymerase at 95 °C for 10 min followed by 40 cycles of a 15 s denaturation at 95 °C followed by a 1 min annealing-extension step at 60 °C.

### Determination of quantitative real-time PCR assay

To quantify the number of copies of *A. phagocytophilum* 16S rRNA and for *Babesia* spp. 18S rRNA genes in ticks, an absolute qPCR was used. For better estimation, we compared qPCR results based on circular plasmids and linearized plasmids prepared by digesting the appropriate plasmid with restriction endonuclease BamHI (Thermo Scientific, USA), and purified using the Clean-up kit (A&A Biotechnology, Gdynia, Poland). Numbers of plasmid molecules were calculated based on the size of recombinant plasmids. A serial standard dilution of the particular standards was used in single reactions.

*Anaplasma phagocytophilum*—tenfold dilutions were developed from the circular and linearized control plasmids pJet1-Aph16S in initial concentration of 1.59 × 10^10^ and 3 × 10^7^ copies/µL, respectively.

*Babesia* spp.—tenfold dilutions were developed from the circular and linearized control plasmids pJet1-Bab18S in initial concentration 2.23 × 10^10^ and 3 × 10^8^ copies/µL, respectively.

All dilutions were made in 0.1 % BSA. Reaction mixtures consisted of 10 µL of the Real-Time 2× PCR Mastermix Probe, 0.4 µL of Aph16S-f and 0.4 µL of Aph16S-r (or Bab18S-f and Bab18S-r) primers (10 µM), 0.2 µL of the Aph16Sp (or Bab18S-p) probe (10 µM), 8 µL of water and 1 µL of the pJet1-Aph16S (or pJet1-Bab18S) plasmid.

Cycling conditions were as follows: 95 °C for 3 min, followed by 40 cycles of a 15-s denaturation at 95 °C, followed by a 1 min annealing-extension step at 60 °C. The resulting standard curves were assessed by analysing them in triplicate in three independent qPCR tests to determine efficiency of amplification, and the lower limit of *A. phagocytophilum* and *Babesia* spp. gene copies detection.

### Differentiation of *Babesia* species

To differentiate the species of real-time PCR-detected “large” *Babesia,* a nested PCRs were performed with outer primers 5-22F and 1661R, which amplify nearly full-length *Babesia* 18S rRNA gene (~1.7-kb) (Birkenheuer et al. 2003) and three pairs of inner primers. Div_up/Div_down and EU1_up/EU1_down (Hilperthschauser et al. 2006) amplified 353 and 362 bp fragments specific for *B. divergens*-like piroplasms (*B. divergens, B. capreoli, Babesia* sp. CH1) and *B. venatorum* (*Babesia* sp. EU1). An inner primer pair 455-479F and 793-772R was designed to amplify an approx. 340 bp fragment from *B. canis* (Birkenheuer et al. 2003).

Amplifications were carried out in the GeneAmp^®^ PCR System 9700 (Applied Biosystem 850, Foster City, CA, USA). The conditions of PCR were as already described (Birkenheuer et al. 2003; Hilperthschauser et al. 2006). Obtained nested PCR products were analysed after electrophoresis in 2 % agarose gel stained with Midori Green DNA Stain (Nippon Genetics Europe).

### Determination of variants of *Anaplasma phagocytophilum*

Nested PCR targeting a 546-bp fragment of the *A. phagocytophilum* 16S rRNA gene was performed in reaction mixtures as described above, under PCR condition described by Massung et al. (1998). All positive samples were sequenced bidirectionally with an ABI 310 Genetic Analyzer (Applied Biosystems, Foster City, CA, USA) and analyzed by using BLASTn (www.ncbi.nlm.nih.gov.blast) analysis of GenBank sequences to determine variants of the gene.

### Statistical analysis

All statistical analyses were performed with the Statistica 10.0. software package (2011) (StatSoft, Inc., Tulsa, OK,USA) (www.statsoft.com). The χ^2^ test was used (with Fisher’s exact test, Cochran test and Yates correction when needed) for qualitative variables. Differences at *p* values ≤0.05 were considered significant.

The effect of relative density (RD) of ticks on the *Babesia* spp. and *A. phagocytophilum* prevalence was investigated with logistic regressions by using MedCalc statistical software (www.medcalc.org/); *p* < 0.05 was regarded as significant.

## Results

**Fig. 2:**
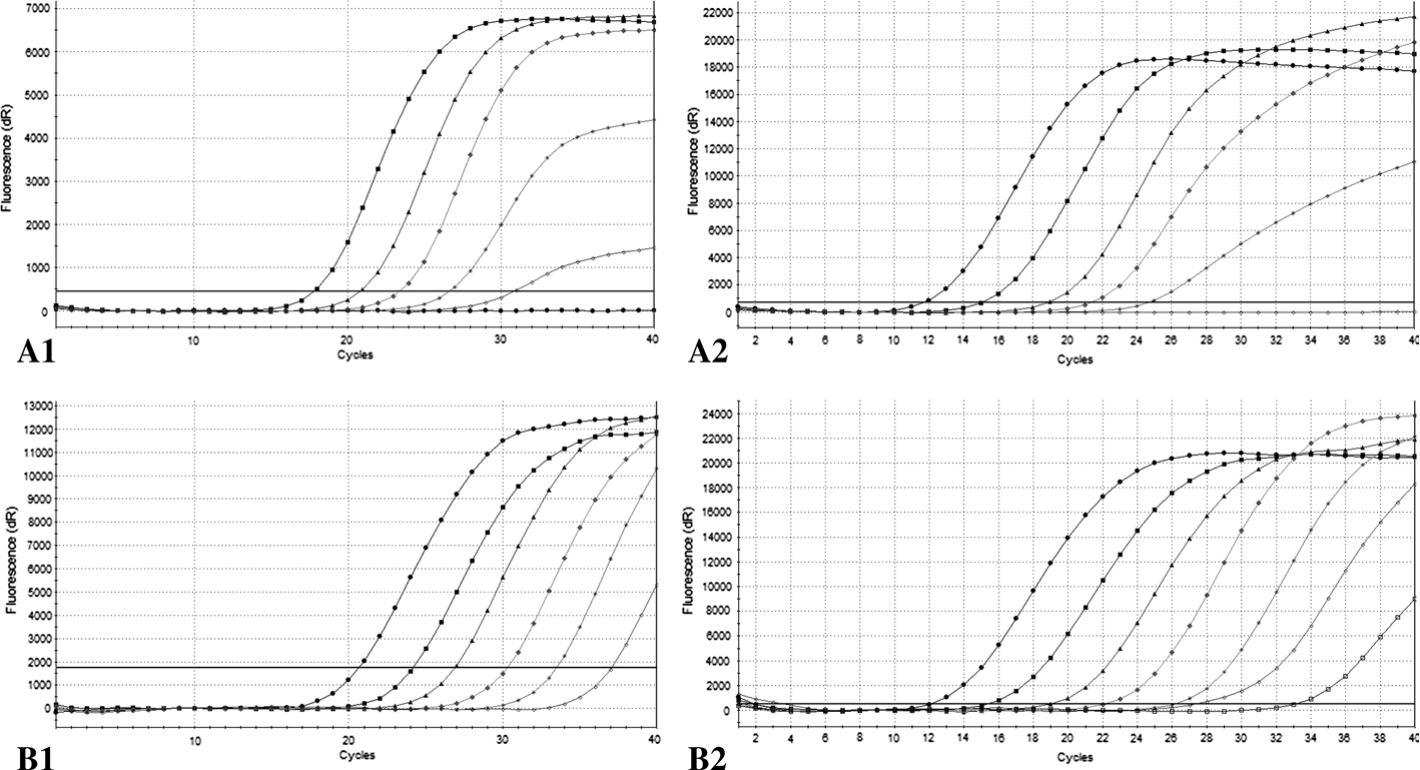
Amplification plots showing the analytical sensitivity of the real-time PCR assay for *Anaplasma phagocytophilum* (**A1**; **A2**) and *Babesia* sp. (**B1**; **B2**). The results from the amplification of tenfold serial dilutions of the *circular* (**A1**) and linearized control plasmids (**A2**) pJet1-Aph16S in initial concentration of 1.59 × 10^7^ copies/µL and 3 × 10^7^ copies/µL, respectively. (Ct values: 17.85, 20.86, 23.46, 26.69, 30.87 and 11.71, 14.70, 17.85; 21.25, 24.30). The results from the amplification of tenfold serial dilutions of the *circular* (**B1**) and linearized control plasmids (**B2**) pJet1-Bab18S in initial concentration 2.23 × 10^7^ copies/µL and 3 × 10^8^ copies/µL, respectively. (Ct values: 20.47, 23,94, 26.69, 30.07, 33.31, 36.86 and 11.71, 14.62,17.77, 21.47, 25.01, 28.83, 32.38)

A duplex real-time PCR assay amplifying the specific fragments of the 16S rRNA gene of *A. phagocytophilum* and the 18S rRNA gene of “large” *Babesia* spp. was designed for simultaneous qualitative detection of target pathogens in *I. ricinus* ticks. Then, to estimate the abundance of microorganisms in tick specimens, the standard curves in absolute qPCR were generated, yielding a linear relationship between Ct and the log_10_ of the starting quantities standard plasmids. Both the circular and linearized standard plasmids were used in single reactions and were found to have significant different threshold cycle numbers (Ct) (*p* < 0.0010). In case of *A. phagocytophilum,* PCR with circular form as a template gave 5.44–6.14 more of the threshold cycle number than did linear standards, while in case of *Babesia* sp. 4.48–6.17, respectively (Fig. 2). The slopes for all four standard curves ranged from −3.174 to −3.493, with an R^2^ of 0.992–1.000. The calculated efficiency varied between 93.3 and 106.6 %.

The lower limit of *A. phagocytophilum* and *Babesia* spp. genes copies detection for both types of standard (circular vs linear) plasmids was 1.59 × 10^3^ versus 3 × 10^3^ and 2.23 × 10^2^ versus 3 × 10^2^, respectively.

Significantly different numbers of copies of 16S rRNA and 18S rRNA genes were estimated for positive tick samples based on circular and linear standard plasmids (*p* < 0.001). The estimates *A. phagocytophilum* from the circular pJet1-Aph16S were 28.7 ± 1.2 times higher than from its linearized form, whereas using circular pJet1-Bab18S as the standard resulted in an estimate of 5.1 ± 1.1 times more copies of *Babesia* spp. when compared with digested plasmid DNA standard. Due to this significant overestimation, in this paper we present results of qPCR calculated on the basis of linearized standard curves as much more reliable.

Altogether 1875 *I. ricinus* ticks, including 791 nymphs (42.2 %) and 1084 adults (57.8 %) (524 females and 560 males), were collected. 757 ticks (450 adults and 307 nymphs) were caught in the urban area while 1118 (634 adults and 484 nymphs) in the rural environment (Table 2). All of them were tested for the presence of *A. phagocytophilum* and *Babesia* spp. by the duplex real-time PCR and total of 3.4 % tested positive for one of these pathogens. However, we cannot exclude the possibility that both species might be present at a lower prevalence below our detection limits: 1.59 × 10^3^ for *A. phagocytophilum* and 2.23 × 10^2^ for *Babesia* spp. Therefore our results represents rather minimal infectivity values, although in case of *A. phagocytophilum* results of preliminary studies showed that the detection sensitivity of our primers and probe set was comparable with the set designed by Courtney et al. (2004) and with nested PCRs (data not shown). Co-infection of both pathogens was not observed. The infection rate of *I. ricinus* collected in the urban environment was at least 6.2 % being significantly higher (*p* < 0.05) than in the rural area—1.5 %. Significantly fewer nymphs (1.1 %; *p* = 0.001) were infected than adults (5.1 %), while females and males showed a comparable infection level (5.3 vs. 4.8 %) (Table 2). *Babesia* spp. was the predominant pathogen group and was detected in a minimum of 2.5 % of all ticks. The minimal overall infection level of *A. phagocytophilum* was 2.8 times lower—0.9 %.

**Table 2:** The minimal prevalence of *Babesia* spp. and *Anaplasma phagocytophilum* in *Ixodes ricinus* collected in forested urban and rural areas in Pomeranian voivodeship, northern Poland

Collection site	*Ixodes ricinus*				
	Stage	No. tested	No./% infected		
			*Babesia* spp.	*A. phagocytophilum*	Total
*Urban environment*					
(A) Gdynia–Kolibki	Female	89	11/12.4	0/0	11/12.4
	Male	110	6/5.5	2/1.8	8/7.3
	Subtotal	199	17/8.5	2/1.0	19/9.5
	Nymphs	168	3/1.8	0/0	3/1.8
	Total	367	20/5.4	2/0.5	22/6.0
(B) Sopot	Female	55	5/9.1	2/3.6	7/12.7
	Male	45	3/6.7	4/8.8	7/15.5
	Subtotal	100	8/8.0	6/6.0	14/14.0
	Nymphs	7	0/0	1/14.3	1/14.3
	Total	107	8/7.5	7/6.5	15/14.0
(C) Gdańsk–Jaśkowa Dolina	Female	75	2/2.7	1/1.3	3/4.0
	Male	76	2/2.6	3/3.9	5/6.6
	Subtotal	151	4/0.4	4/2.6	8/5.3
	Nymphs	132	2/1.5	0/0	2/1.5
	Total	283	6/2.1	4/1.4	10/3.53
Subtotal	Female	219	18/8.2	3/1.4	21/9.6
	Male	231	11/4.8	9/3.9	20/8.7
	Subtotal	450	29/6.4	12/2.7	41/9.1
	Nymphs	307	5/1.6	1/0.3	6/1.95
	Total	757	34/4.5	13/1.7	47/6.2
*Rural environment*					
(D) Kartuzy	Female	14	0/0	0/0	0/0
	Male	20	0/0	1/5.0	1/5.0
	Subtotal	34	0/0	1/2.9	1/2.9
	Nymphs	83	0/0	0/0	0/0
	Total	117	0/0	1/0.9	1/0.9
(E) Borzestowska Huta	Female	153	1/0.7	3/2.0	4/2.6
	Male	157	2/1.3	0/0	2/1.3
	Subtotal	310	3/1.0	3/1.0	6/1.9
	Nymphs	233	1/0.4	0/0	1/0.4
	Total	543	4/0.7	3/0.6	7/1.3
(F) Mirachowo	Female	63	1/1.6	0/0	1/1.6
	Male	61	2/3.3	0/0	2/3.3
	Subtotal	124	3/2.4	0/0	3/2.4
	Nymphs	61	0/0	0/0	0/0
	Total	185	3/1.6	0/0	3/1.6
(G) Sulęczyno	Female	75	2/2.7	0/0	2/2.7
	Male	91	2/2.2	0/0	2/2.2
	Subtotal	166	4/2.4	0/0	4/2.4
	Nymphs	107	2/1.9	0/0	2/1.9
	Total	273	6/2.2	0/0	6/2.2
Subtotal	Female	305	4/1.3	3/1.0	7/2.3
	Male	329	6/1.8	1/0.3	7/2.1
	Subtotal	634	10/1.6	4/0.6	14/2.2
	Nymphs	484	3/0.6	0/0	3/0.6
	Total	1118	13/1.2	4/0.4	17/1.5
Total	Female	524	22/4.2	6/1.1	28/5.3
	Male	560	17/3.0	10/1.8	27/4.8
	Subtotal	1084	39/3.6	16/1.5	55/5.1
	Nymphs	791	8/1.0	1/0.1	9/1.1
	Total	1875	47/2.5	17/0.9	64/3.4

Both pathogens were detected in A–C locations in the Tri-City, and at sites E, F, G and D, E, respectively, of four locations in the KLP. In total, study site B showed a significantly higher prevalence of positive ticks than all other study sites (*p* < 0.002), while site D presented a significantly lower prevalence (*p* < 0.002). Locally, in the Tri-city, the percentage of ticks tested positive for babesiae (2.1–6.5 %) differed significantly at particular sites (*p* = 0.006–0.011), while there were no statistical differences observed in the infection rates of ticks collected in the rural area (*p* > 0.05).

In the Tri-city sites, percentage of babesiae- and rickettisiae-positive ticks was significantly lower in nymphs (1.6 and 0.3 %. respectively) than in adults (6.4 and 1.7 %, respectively) (*p* = 0.001), with the prevalence statistically compared in males and females (*p* = 0.09–0.1). In the rural woodlands there were no statistical differences between adult ticks (*p* = 0.2–0.6) and in adults compared to nymphs (*p* = 0.08–0.1; Table 2).

Analysis of seasonal dynamics of infected ticks showed that variations among the collection months in both studied areas were not significant (*p* > 0.05), with one exception. In mature ticks, the minimal infection rate with *Babesia* spp. in June (3.8 %) increased significantly compared to April (0.7 %) (*p* = 0.047).

According to a 5-points scale (Supergon and Karbowiak 2009), the RD of ticks per 1 h of collection of total sampling at particular sites in the Tri-City ranged between high (2°RD) and medium (3°RD), while in the KLP varied from very high (1°RD) to medium (Table 3) (Stańczak et al. 2012). The percentage of *I. ricinus* tested positive for *Babesia* spp. was significantly higher at sites with 3º (OR 3.3937; *p* = 0.029) and 2°RDs (OR 5.5148; *p* = 0.0014) than at the site with 1°RD. Locally, a similar trend was observed in the city sites of medium (B, C) (3.6 %) (OR 5.0173; *p* = 0.0047) and high RD of ticks (A) (5.4 %) (OR 7.3978; *p* = 0.0002) as well as in the rural site of high RD (G) (2.2 %) (OR = 2.8883; *p* = 0.098) (Table 3). Due to low prevalence of *A. phagocytophilum* in ticks, odds ratios were interpretable as relative risks (RR). The infection prevalence (1.7 %) was significantly higher in sites with 3°RD of ticks (B, C, D, F) (RR 3.1387; *p* = 0.075).

**Table 3:** Minimal % of *Ixodes ricinus* infected with *Babesia* spp. and *Anaplasma phagocytophilum* in relation to a relative density (RD)^a^ of ticks

RD (risk level)	Collection site	*Babesia* spp.	*A. phagocytophilum*
3°: 11–25 (medium)	B	7.5	6.5
	C	2.1	1.4
	Subtotal	3.6	2.8
	D	0.0	0.9
	F	1.6	0.0
	Subtotal	1.0	0.3
	Total	2.5 (OR 3.3937, 95 % CI 1.1352–10.1452, *p* = 0.029)	1.7 (RR 3.1387, 95 % CI 0.8902–11.0673, *p* = 0.075)
(2°) 26–50 (high)	A	5.4	0.5
	G	2.2	0.0
	Total	4.1 (OR 5.5148, 95 % CI 1.9367–15.7038, *p* = 0.0014)	0.3 (RR 0.5656, *p* = 0.53)
(1°) >50 (very high)	E	0.7	0.6

*OR* odds ratio, *RR* relative risk

^a^ *RD*—number of ticks per one person per one hour of collection of total sampling at particular sites (Supergon and Karbowiak 2009)

In conclusion, logistic regression analysis of the relationship between the probability of infection in ticks and location, stage, month and sex of ticks showed a statistically significant negative correlation for nymphs and for ticks from a rural environment. In case of *Babesia* spp., the regression coefficient was *b* = −0.74 (*p* = 0.003, OR = 0.48, 95 % CI 0.29–0.78) and −0.63, respectively. For *A. phagocytophilum*, *b* = −1.61 (*p* = 0.02, OR 0.20, 95 % CI 0.05–0.7) and −0.78 (*p* = 0.007, OR 0.46, 95 % CI 0.26–0.81).

### Results of qPCR

The estimated average number of 18S rRNA gene copies of *Babesia* spp. per positive tick sample was 2.55 × 10^5^ ± 1.04 × 10^6^ (Table 4). Overall, average copy numbers detected per female tick in both sampling areas were 3.8-fold higher than in male ticks and those detected in adult specimen were ~20 times higher than in nymphs (*p* = 0.001) (Table 4). In the Tri-City, however, these differences were not significant (*p* = 0.71), while among *I. ricinus* collected in the KLP, a significant difference was observed between females and nymphs (*p* = 0.03; Table 4).

**Table 4:** Results of the qPCR assay (mean copy number per infected tick ± SD) targeting the 18S rRNA gene of “large” *Babesia* spp. in *Ixodes ricinus* ticks

	Urban area	Rural area	Total
Females			
Mean	4.90 × 10^5^ ± 1.69 × 10^6^	3.53 × 10^5^ ± 1.56 × 10^5^	4.43 × 10^5^ ± 1.48 × 10^6^
Range	6.05 × 10^3^–6.70 × 10^6^	1.97 × 10^5^–5.21 × 10^5^	6.05 × 10^3^ ± 6.70 × 10^6^
Median	1.62 × 10^4^	3.46 × 10^5^	1.27 × 10^4^
Males			
Mean	4.67 × 10^4^ ± 8.04 × 10^5^	2.30 × 10^5^ ± 3.36 × 10	3.05 × 10^5^ ± 2.23 × 10^5^
Range	6.43 × 10^3^–2.17 × 105	1.30 × 10^4^–8.82 × 10^5^	6.43 × 10^3^–8.83 × 10^5^
Median	1.32 × 10^3^	2.20 × 10^5^	1.53 × 10^4^
Adults—total			
Mean	3.15 × 10^5^ ± 1.32 × 10^6^	2.80 × 10^5^ ± 2.74 × 10^5^	3.05 × 10^5^ ± 1.14 × 10^6^
Range	6.05 × 10^3^–6.70 × 10^6^	1.3 × 10^4^–8.83 × 10^5^	6.05 × 10^3^–6.70 × 10^5^
Median	2.09 × 10^3^	2.20 × 10^5^	1.46 × 10^4^
Nymphs			
Mean	1.54 × 10^4^ ± 157 × 10^4^	1.55 × 10^4^ ± 1.29 × 10^4^	1.54 × 10^4^ ± 1.37 × 10^4^
Range	6.13 × 10^3^–3.78 × 10^4^	5.33 × 10^3^–3.00 × 10^4^	6.13 × 10^3^–3.37 × 10^4^
Median	1.53 × 10^4^	1.13 × 10^4^	1.25 × 10^4^
Total			
Mean	2.69 × 10^5^ ± 1.21 × 10^6^	2.18 × 10^5^ ± 2.64 × 10^5^	2.55 × 10^5^ ± 1.04 × 10^6^
Range	6.05 × 10^3^– 6.7 × 10^6^	5.33 × 10^3^–8.33 × 10^5^	6.05 × 10^3^–6.7 × 10^6^
Median	3.02 × 10^3^	1.41 × 10^5^	1.21 × 10^4^

Although variable copy numbers of *Babesia* spp. (1.11 × 10^5^–7.43 × 10^5^) were estimated for ticks collected in a particular collection month, with the peak in April, followed by the lower one in June (1.54 × 10^5^), these differences were not significant (*p* = 0.20).

Seventeen tick samples tested positive for *A. phagocytophilum* contained an average 16S rRNA copy number of 3.39 × 10^5^ (range 2.53 × 10^3^–9.7 × 10^5^; median 3.54 × 10^5^) per specimen (Table 5). In total, an estimated mean load of the pathogen in female samples was 1.94 × 10^5^ and in male samples was 4.27 × 10^5^, but differences were not significant (*p* = 0.48). The average number of copies per one tick in the urban area was ~10 times higher than in the rural one, but this difference was also not significant (*p* = 0.10). However, this comparison may be not adequate due to the low number of infected *I. ricinus* in the KLP (n = 4) in comparison to the Tri-City. For all polled sites, *A. phagocytophilum* abundance peaked in May (2.27 × 10^5^). The number of ticks infected with *A. phagocytophilum*, however, was too small to draw clear conclusions.

**Table 5:** Results of the qPCR assay (mean copy number per infected tick ± SD) targeting the 16S rRNA gene of *Anaplasma phagocytophilum* in *Ixodes ricinus* ticks

	Urban area	Rural area	Total
Females			
Mean	2.82 × 10^5^ ± 4.6 × 10^5^	1.47 × 10^4^ ± 1.97 × 10^5^	1.94 × 10^5^ ± 3.84 × 10^5^
Range	2.04 × 10^4^–9.7 × 10^5^	1.50 × 10^4^ –3.75 × 10^5^	2.04 × 10^4^–9.7 × 10^5^
Median	6.92 × 10^4^	5.99 × 10^4^	6.92 × 10^4^
Males			
Mean	4.71 × 10^5^ ± 7.46 × 10^5^	3.34 × 10^4^*	4.27 × 10^5^ ± 7.17 × 10^5^
Range	2.53 × 10^3^–7.07 × 10^5^	nc	2.53 × 10^3^–7.07 × 10^5^
Median	1.09 × 10^5^	nc	1.09 × 10^5^
Nymphs			
Mean	Below detection limit		
Total			
Mean	2.03 × 10^5^ ± 6.76 × 10^5^	1.95 × 10^4^ ± 1.85 × 10^5^	3.39 × 10^5^ ± 6.09 × 10^5^
Range	2.53 × 10^3^–9.7 × 10^5^	1.50 × 10^4^–3.75 × 10^5^	2.53 × 10^3^–9.7 × 10^5^
Median	2.90 × 10^5^	1.97 × 10^4^	3.54 × 10^5^

### *Babesia* species and *Anaplasma phagocytophilum* 16S rRNA gene variants

*Babesia* positive tick samples were assigned to the different species by nested PCR assay with primers specific for *B. venatorum* (former EU1), *B. divergens*-like and *B. canis.* The predominant species both in the rural as well as in the urban areas was *B. venatorum* (68 %), followed by *B. canis* (27 %) and *B. divergens*-like (5 %). The first two species were noted both in the urban and rural areas, while the latter one only at site E.

To determine variants of *A. phagocytophilum,* positive samples were sequenced. Alignment of 11 obtained 514–524 bp fragments of 16S rRNA gene revealed four variants with 99.8–100 % identity to each other. None of them shared 100 % homology with compared sequences isolated from humans (GenBank acc. no.: U02521; AF093789; AY833407; AY886761; KF111754). However, the most prevalent variant 1 (n = 7) showed 100 % identity to homologous sequences deposited in GenBank: JX173651 (dog blood, Germany), HM138366 (cat blood, Czech Republic) and JN181064 (*I. ricinus,* Lithuania). The consensus sequence was deposited to GenBank under accession no. KP245905. Variant 2 (acc. no. KP245906) (n = 1) was 100 % homologous to the following sequences of *A. phagocytophilum:* JN181070 (*I. ricinus* from the European robin, Norway), JN181073 (*I. ricinus* from a red fox, Norway) and HQ629923 (*I. ricinus,* Estonia). Variant 3 (acc. no. KP245907) (n = 2) was identical with AF384214—*Ehrichia phagocytophila* (roe deer, Switzerland) and with FJ788513 (*I. ricinus* from a roe deer, Germany). Variant 4 (acc. no KP245908) (n = 1) showed 100 % similarity with JN181069 (*I. ricinus,* Lithuania), KC800983 (spleen of an elk, Sweden) and JQ06324 (cotton rat; FL, USA). Of four variants, 1 and 3 were detected in the urban area. Both occurred in ticks collected at location A, while at site B only variant 1 was found (n = 6). Three variants—2, 3 4, were noted in the rural environment. The first two originated from the ticks collected at site E, while the latter was detected in a tick collected at site D.

## Discussion

In this study, we used a duplex real-time PCR assay to detect simultaneously the presence of *Babesia* spp. and *A. phagocytophilum* in field-collected *I. ricinus* ticks. As it has been observed that multiplexing qPCR assays might increase the likelihood of compromising the efficiency of individual target assays probably due to competitive amplifications and/or interaction of the fluorophores (Bialasiewicz et al. 2007), we chosen the fluorophores and quenchers based on their compatibility in one multiplex assay under the same PCR parameters.

Both singleplex assay (a single set of the primer and probe in reaction mixtures) and duplex assay (reaction mixtures containing primer and probe sets for the two genes targeted) was assessed and when compare, the assays performed the same (data not shown). Although the detection sensitivity was not as high as expected, percentage of questing ticks tested positive for *A. phagocytophilum* and *Babesia* spp. in both natural (the Kashubian Landscape Park) and urban (Tri-City agglomeration) environments (0.9 and 2.5 %, respectively) was comparable to those noted in Belarus (Reye et al. 2013), Lithuania, Norway (Radzijevskaya et al. 2008) and Luxemburg (Reye et al. 2010).

The results of regression analysis showed that the probability of infection of ticks with both pathogens decreases significantly in nymphs and in the rural environment. The observation that significantly less nymphs (*p* = 0.0010) tested positive than adult *I. ricinus* while females and males showed a comparable infection level is in agreement with the other reports (Grzeszczuk 2006; Silaghi et al. 2008; Reye et al. 2010; Mysterud et al. 2013; Welc-Falęciak et al. 2014). Significantly higher prevalence of both tick-borne pathogens (TBPs) in urban compared with natural environments was also reported by Welc-Falęciak et al. (2014) in Warsaw (CE Poland) and by Silaghi et al. (2008) in Munich, Germany. Intriguingly, in our study the highest percentage of positive-testing ticks (14 %) was observed in Sopot-Karlikowo, the small area enclosed by roads and houses and separated from the forests of the TCLP. Although ticks were not numerous there at least 7.5 % of them were infected with *Babesia* spp. and 6.5 % with *A. phagocytophilum.*

In total, we observed some variation between the tick density and tick infection rates (*p* = 0.0287). In The Netherlands, Coipan et al. (2013) also detected a slight negative correlation between the density of questing *I. ricinus* and the infection prevalence with *Babesia* spp. and no correlation between these two variables in the case of *A. phagocytophilum.* On the other hand, Welc-Faleciak et al. (2014) observed that the prevalence of *A. phagocytophilum* did not differ statistically between ticks from low- and high tick-density forests in central and north-eastern Poland.

Reports concerning the seasonal variations of tick infection rates with *Babesia* spp. and *A. phagocytophilum* are not numerous. In Luxemburg, tick infection rates with both pathogens were low in July and August and significantly increased in September. In Norway, a markedly higher infection rate was observed in May compared to August (Mysterud et al. 2013), while in The Netherlands the annual prevalence of *A. phagocytophilum* was not seasonal while *Babesia* spp. showed highest prevalence in ticks in a time period corresponding to October (Coipan et al. 2013). In our study, bimodal pattern of seasonality (May and July) observed for ticks infected with *Babesia* spp. in the urban environment corresponded with the peaks of tick activity in this area, while in the rural environment a single peak noted in June followed the spring peak of *I. ricinus* activity (May) observed in the KLP (Stańczak et al. 2012). For *A. phagocytophilum* bimodal patterns were noted in both areas investigated with peaks in April and June prior to increased activity of questing tick. The higher infection level observed in April–may suggests that ticks acquired infection with blood meals taken in the previous year.

Only a few studies have taken the load of *Babesia* spp. and *A. phagocytophilum* or other TBPs in infected ticks or their hosts into account. The best known is the paper by Courtney et al. (2004) who developed a duplex real time PCR for simultaneous detection of *B. burgdorferi* and *A. phagocytophilum.* They detected both pathogens also in the field collected *I. scapularis*, but did not give information about the load of pathogens in the tested ticks. Other researchers (Radzijevskaya et al. 2008; Silaghi et al. 2008; Rosef et al. 2009; Mysterud et al. 2013) used the same method to analyse prevalence of these pathogens in *I. ricinus* but also did not carry out detailed quantification studies. In case of *Babesia* spp. a majority of investigations focused on developing a specific qPCR assay designed to detect DNA in human and animal blood samples using a primer and probe combination that targets the 18S rRNA gene of *B. microti,* the dominant babesiosis pathogen in the USA (Wang et al. 2010; Teal et al. 2012). Recently Švehlová et al. (2014) have screened ticks for the presence of *R. helvetica* by using a qPCR assay targeting a fragment of 23S rRNA gene and the pathogen loads was from 2.73 × 10^4^ to as many as 4.23 × 10^13^ copy number per tick.

We have attempted to estimate the approximate number of copies of the target genes of *A. phagocytophilum* and *Babesia* spp. in templates obtained from questing *I. ricinus.* For this purpose we designed absolute qPCR targeting a fragment of a 16S rRNA gene, which is present in a single copy per *Anaplasma* spp. genome (Rurangiwra et al. 2002) and a fragment of a 18S RNA gene as the sensitive molecular target in the case of babesiae. It is known that *B. microti* contains two and *B. gibsoni*, the other “small *Babesia”,* five copies of this gene. However, *B. divergens*, *B. venatorum* and *B. canis* belong to “large” *Babesia* species of an unknown number of 18S rRNA copies (Brayton et al. 2007; Cornillot et al. 2012), which makes quantification in field-collected samples less accurate. Recently Hou et al. (2010) have documented that different structural types of DNA used as the plasmid standards may seriously affect the quantification accuracy of qPCR. Using as a model a *pcna* gene of marine microalgae, the authors compared circular and linearized plasmid DNA and observed that PCR with circular form as a template gave 2.65–4.38 more of the threshold cycle number than did linear standards and yielded an estimate of 7.77 copies of *pcna* per genome, in comparison to the highly accurate 1.02 copies given by linear standard. The results obtained by us also demonstrated significant differences in the Ct numbers and in the abundances of *A. phagocytophilum* and *Babesia* spp. when different control plasmids were employed. Numbers of detected copies of 16S rRNA and 18S rRNA were 28.7 and 5.1 times higher when the circular plasmid standards were used. Thus, linear plasmids seem more reliable and should be recommended in qPCR reactions. Although we observed differences in copy numbers in relation to tick density, sampling areas and months of collection, they were insignificant. In the Tri-city, the load of pathogens in positive nymphs was significantly lower than in adults (*p* = 0.001), and comparable in males and females (*p* = 0.09–0.1). In the rural woodlands there were no statistical differences between adult ticks (*p* = 0.2–0.6) and in adults compared to nymphs (*p* = 0.08–0.1). The low number of positive ticks prevented reaching a definite conclusion.

The dominant *Babesia* sp. was *B. venatorum,* which was first detected in native ticks in the Tri-City municipal forests in 2008 (Cieniuch et al. 2009). Recently, it has also been detected in the Bielański Forest in Warsaw (CE Poland), where it was the only babesial species detected in *I. ricinus* (Welc-Falęciak et al. 2012). Moreover, *B. venatorum* also dominated in Bavarian public parks, Germany (Schorn et al. 2011a). Our results confirm that this species is well established in tick populations in the urban environment and should be considered a potential threat to humans. The three European *B. venatorum*-infected patients were men who undergone splenectomies and were at risk group for babesiosis (Herwaldt et al. 2003; Haselbarth et al. 2007). However, recently 33 confirmed cases and 16 probable cases of babesiosis caused by this species have been reported in China and none of infected person had a splenectomy or had received a blood transfusion (Sun et al. 2014, Jiang et al. 2015). It suggests that *Babesia* sp. can cause infections also in healthy and immunocompetent persons.

We also confirmed the possibility of *I. ricinus* infection with *B. canis,* which was previously noted by us in 0.2 and 0.1 % ticks collected in urban and rural areas, respectively (Cieniuch et al. 2009). This is an interesting finding, because *Dermacentor reticulatus*, a main vector of canine babesiosis in Poland, rarely is noted in the Pomeranian region (Fryderyk 1998; Kadulski and Izdebska 2009).

Of the four genetic variants of the partial 16S rRNA of *A. phagocytophilum* detected in *I. ricinus*, none equaled in the amplified part the human pathogenic prototype variant (GenBank U02521). However, sequences of all of them were 100 % homologous to other sequences already deposited in GenBank. The most prevalent variant 1 has been already detected for example in ticks, dogs and cats (Dyachenko et al. 2013, Paulauskas et al. 2012, Hulinska D, unpublished). Variant 2 was found in *I. ricinus* feeding on birds and red foxes (Paulauskas et al. 2012). Variant 3 has been previously detected in roe deer and in ticks feeding on them (Liz et al. 2002, Heyl et al., unpublished) while variant 4 was found in *I. ricinus* (Paulauskas et al. 2012), an elk and, interestingly, showed 100 % identity with sequence from a cotton rat form Florida, USA (Clark 2012).

In Poland, roe deer (*Capreolus* *capreolus*) and red deer (*Cervus* *elaphus*) seem to be greatly involved in the wild cycle of *B. venatorum, B. divergens* and *A. phagocytophilum* (Adamska and Skotarczak 2007, Michalik et al. 2009, Stańczak and Michalik, unpublished). We suppose that cervids play an important role in the maintenance of these microorganisms in the TCLP and in the KLP forests as they freely migrate from one forest complex to another one. In the intra-city site Sopot-Karlikowo, where the highest percentage of ticks infected with *B. venatorum* and *A. phagocytophilum* was noted, *I. ricinus* can be brought by red foxes which appear to be increasing in urban environments. In Germany, at least three *Babesia* species: *B. microti, B. venatorum* and *B. capreoli* were found in *Ixodes* spp. parasitizing these animals (Najm et al. 2014). Also birds can introduce infected ticks (Hasle et al. 2011; Mărcuţan et al. 2014). Moreover, the role of dogs in maintenance of *A. phagocytophilum* has been discussed (Schorn et al. 2011b), although some authors suggests (Stuen et al. 2013) that dogs host mainly adult ticks and therefore cannot effectively contribute to the life cycle of *A. phagocytophilum*, as transovarial infection does not seem to occur.

## Conclusion

We have developed a real-time duplex PCR assay for the simultaneous detection of *Babesia* spp. and *A. phagocytophilum* that showed sensitivity and specificity comparable to that of traditional nested PCR used in our previous investigations. We also confirmed that for the quantification of microorganism abundance in infected ticks linear plasmid standard should be recommended to generate the standard curve in an absolute qPCR as using circular standard resulted in serious overestimation of copies of targeted genes.

The results of the study confirm the presence of *A. phagocytophilum* and *Babesia* spp. in populations of *I. ricinus* in rural and urban environments with the prevalence of these pathogens significantly higher in municipal forests and greenspaces. The determined overall tick infection rate, however, was low and it allows to assume that the disease risk of people and/or their companion animals when coming into contact with ticks is also low.

Nevertheless, local populations, tourists and physicians should be aware of the potential possibility of *Babesia* spp. and *A. phagocytytophilum* infection especially that the knowledge about human babesiosis and HGA is still unsufficient among the latters. Infection with both pathogens must be considered in differential diagnosis of TBDs.
